# Real-Time Weld Quality Prediction Using a Laser Vision Sensor in a Lap Fillet Joint during Gas Metal Arc Welding

**DOI:** 10.3390/s20061625

**Published:** 2020-03-14

**Authors:** Kidong Lee, Insung Hwang, Young-Min Kim, Huijun Lee, Munjin Kang, Jiyoung Yu

**Affiliations:** 1 Joining R & D Group, Korea Institute of Industrial Technology, 156 Gaetbeol-ro, Yeonsu-Gu, Incheon 21999, Korea; leekid@kitech.re.kr (K.L.); hisman@kitech.re.kr (I.H.); ymkim77@kitech.re.kr (Y.-M.K.); 2 Monisys Co., Ltd., 775, Gyeongin-ro, Yeongdeungpo-Gu, Seoul 07299, Korea; manian12@hanmail.net

**Keywords:** laser vision sensor, camera calibration, deep neural network, gas metal arc welding, weld quality prediction

## Abstract

Nondestructive test (NDT) technology is required in the gas metal arc (GMA) welding process to secure weld robustness and to monitor the welding quality in real-time. In this study, a laser vision sensor (LVS) is designed and fabricated, and an image processing algorithm is developed and implemented to extract precise laser lines on tested welds. A camera calibration method based on a gyro sensor is used to cope with the complex motion of the welding robot. Data are obtained based on GMA welding experiments at various welding conditions for the estimation of quality prediction models. Deep neural network (DNN) models are developed based on external bead shapes and welding conditions to predict the internal bead shapes and the tensile strengths of welded joints.

## 1. Introduction

The chassis of the car is a component that supports all the other parts, including the car body and the powertrain. These parts are exposed to vibration, shock, twist, and other stresses [1]. Therefore, hot-rolled (HR) steel plates (>440 MPa) are generally used on the chassis. These HR steel plates are manufactured with forming processes. Accordingly, the formed steel plates are joined by gas metal arc welding (GMAW) to form the final chassis parts. In general, a welded component is one of the weakest parts in each chassis section because welding arc joints generate metallurgical and structural discontinuities in the chassis [2,3,4]. Therefore, the weld lines in the chassis parts affect the overall performances of the chassis parts. Additionally, the geometry of the weld bead has a dominant influence on the mechanical performance of the chassis parts, that is, on their tensile strengths and fatigue characteristics [5,6,7,8,9]. The bead geometry is composed of five parts, including the weld face, root, toe, leg, and throat. The qualities of these parts are predominantly determined by the type of welding technology, welding condition, welding position, and joint fit-up [7,10]. To monitor the welding quality and secure robust welds, nondestructive test (NDT) technology is required that can measure the external shape of the weld bead (hereafter referred to as the “external bead shape”) and predict its internal shape (hereafter referred to as the “internal bead shape”). 

Visual systems are highly related to the automation of the welding processes, and vision-based monitoring systems have been mainly used and developed for tracking weld seam and inspecting weld quality [11,12,13]. For weld seam tracking, Zou et al. [14,15] designed a laser vision seam tracking system that can determine weld feature points and obtain the three-dimensional (3D) coordinate values of these points in real-time based on the morphological image processing method and continuous convolution operator tracker (CCOT) object tracking algorithm. Zhang et al. [16] designed a weld path autonomous programming system based on laser structured light scanning, and developed algorithms for processing the image data collected by a laser vision sensor (LVS) to obtain the 3D information of the examined workpiece based on multiple segment scanning. Xue et al. [17] proposed a laser-vision-based detection method for a narrow butt joint, whereby a crosshair laser was projected onto the surface of the workpiece, and an auxiliary light source was applied to illuminate the workpiece surface continually. Wu et al. [18] developed an image processing algorithm based on the modified Hough algorithm and applied it to a laser vision system for seam tracking during the GMAW process. In the case of weld bead geometry measurements, Huang et al. [19,20] developed a laser vision system based on the principle of laser triangulation for nondestructive weld quality inspection, which processed images acquired from the vision sensor and analyzed the acquired 3D profiles of the weld to inspect the positions and sizes of the weld defects. Nguyen et al. [21] also developed and implemented a laser-vision-based weld quality inspection system based on the principle of laser triangulation for nondestructive weld measurements and defect detection. This system adopted a sliding vector method for a fast and reliable approach to detect feature points on the laser stripe profile of welds. Ye et al. [22] studied a model-based classification method that used a polynomial model in conjunction with the expectation-maximization (EM) algorithm for weld bead recognition on the 3D bead profile measured by an LVS. Recently, the deep learning algorithm has been intensively applied for the monitoring of the welding process and the prediction of the weld quality owing to its high-performance predictive power. Shin et al. [23] used a deep neural network (DNN) for gas metal arc welding to detect internal weld defects that cannot be easily detected without the use of nondestructive testing (NDT) methods. Zhang et al. [24] established a deep learning framework based on stacked sparse autoencoder (SSAE) to model the relationship between multisensor features and their corresponding welding statuses for real-time monitoring of high-power disk laser welding.

Previous studies had mainly focused on the measurement of external bead shapes, which are important for weld quality evaluation. However, the technology that can measure external bead shapes and estimate the internal bead shapes is required because both bead shape types play important roles in the determination of weld quality. It is also desirable for the monitoring technology to be able to examine weld bead geometry and predict performance indicators, such as tensile strength and geometrical characteristics in real-time to improve the efficiency of the welding process.

This study presents a laser vision system that can measure an external bead shape and predict the internal bead shape and tensile strength of a weld joint in real-time. An LVS based on the principle of laser triangulation was designed and fabricated with a complementary metal-oxide semiconductor (CMOS) sensor and a blue laser at 405 nm. An image processing algorithm was developed and implemented to measure the external bead shape and detect the feature points of the weld bead. A camera calibration method that can reduce measurement errors caused by the rotation of the LVS was also proposed and implemented to cope with the complex motion of the welding robot. To identify the relationship between the external and internal bead shapes, experimental weld bead cross-sectional data were obtained in GMAW experiments using uncoated HR steel plates with a 590 MPa grade, in lap-joint configurations at different welding conditions, including welding position, wire feeding speed, and different gaps between successive steel plates. DNN models were developed for the prediction of the internal bead shapes and the tensile strengths of the weld joints based on external bead shape and welding condition data. The developed laser vision system was experimentally verified.

## 2. Materials and Methods

### 2.1. Laser Vision System

**Figure 1:**
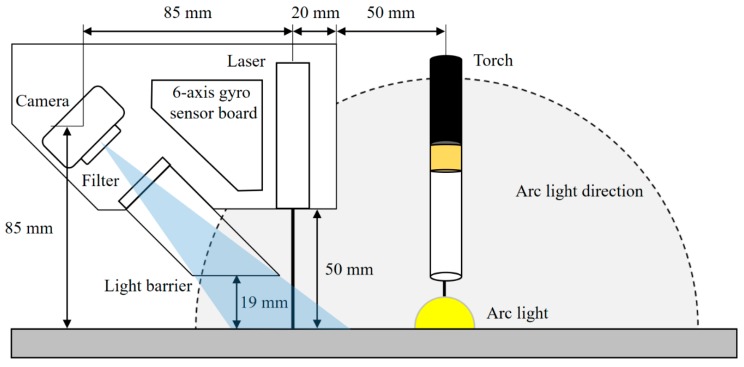
Schematic of a laser vision system.

**Figure 2:**
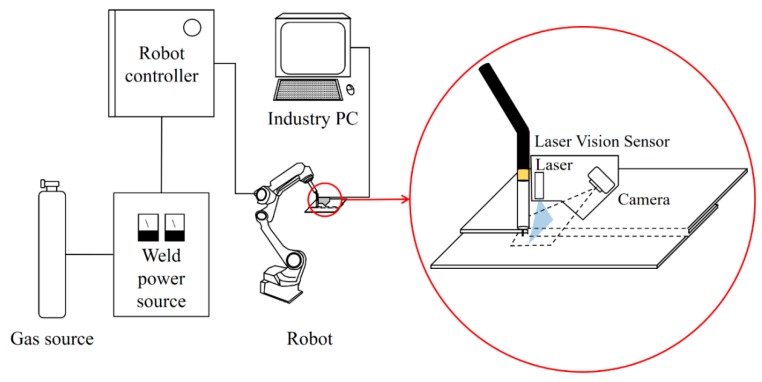
Experimental setup of a welding system with an LVS.

Figure 1 shows the configuration of the laser vision system, including a laser generator, camera, filter, light barrier, and six-axis gyro sensor board. A camera (UI-3271LE-M-GL-VU, IDS Imaging Development Systems GmbH, Obersulm, Germany) that had a CMOS sensor with a resolution of 2056 × 1542 pixels was used. A customized blue-line laser with a power of 50 mW and a wavelength of 405 nm was used, and bandpass (405 nm) and ultraviolet (UV) filters were employed. A motion processing unit (MPU-6050, TDK Corporation, Tokyo, Japan) was used for the six-axis gyro sensor board. A personal computer with an Intel Core i5-8500 processor was used to process acquired images and operate the LVS system with a graphical-user-interface program developed in this study. As shown in Figure 2, the LVS was installed at a position 50 mm above the specimen to receive the reflected laser light from the specimen. The light barrier was designed and installed to (a) prevent arc light from directly entering the camera and to (b) receive only the reflected laser light. The welding experiment was carried out with the use of an industrial robot (IRB 2400, ABB Group, Zurich, Switzerland).

### 2.2. Experimental Procedure

**Figure 3:**
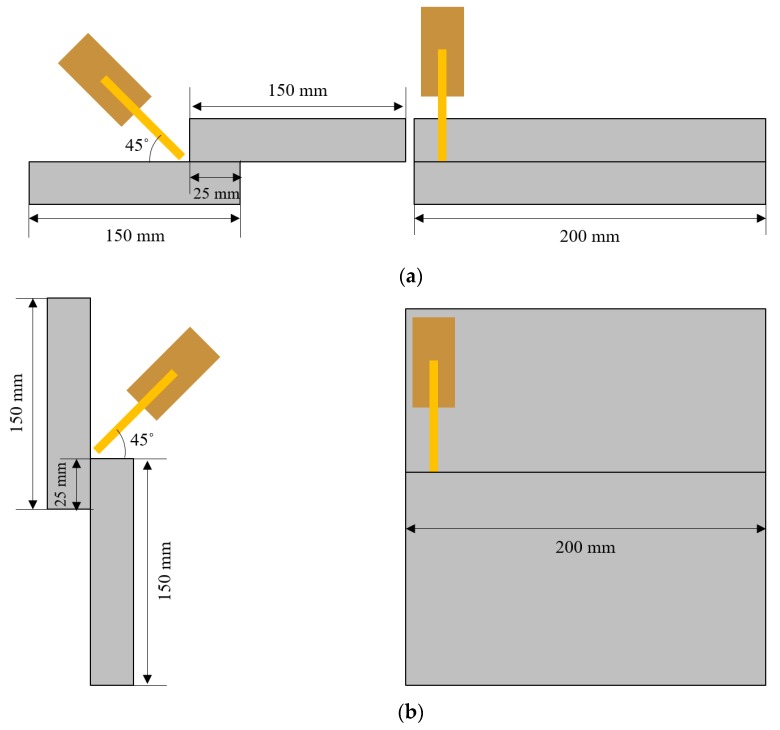
Schematics of the torch position, welding position, and tested workpiece. (**a**) Flat position; (**b**) Horizontal position.

An uncoated HR steel with a thickness of 2.3 mm and ultimate tensile strength of 590 MPa was used in this study. Table 1 shows the chemical composition and mechanical properties of the HR steel plate. An ER70S welding wire with a diameter of 1.2 mm was used, and a gas mixture which consisted of 90% Ar and 10% CO_2_, was used as the shielding gas. Figure 3 shows the schematics of the welding position, torch angle, and the configuration of the test workpiece. As presented in Table 2, a welding experiment was conducted using two welding processes of standard direct current (DC) GMAW and cold metal transfer (CMT) GMAW in the flat position (PA) and horizontal position (PC). We selected a wire feeding speed range that covered various conditions that ranged from the lack of fusion to sufficient welding. Other welding conditions, including welding speed, work angle, travel angle, contact tip-to-work distance (CTWD), and the gap between workpieces, are summarized in Table 2. Welding experiments were carried out with 100 different welding conditions. The external bead shape was measured simultaneously with welding in real-time in all the tested experimental conditions using the LVS shown in Figure 2. Five samples were cut from each welded joint. Two of them were used for weld cross-sectional examinations, while the remaining three samples were used for tensile tests to model the relationship between the external bead shape, internal bead shape, welding parameters, and the tensile strength of the weld.

## 3. Development of an LVS

### 3.1. Image Processing Algorithm

**Figure 4:**
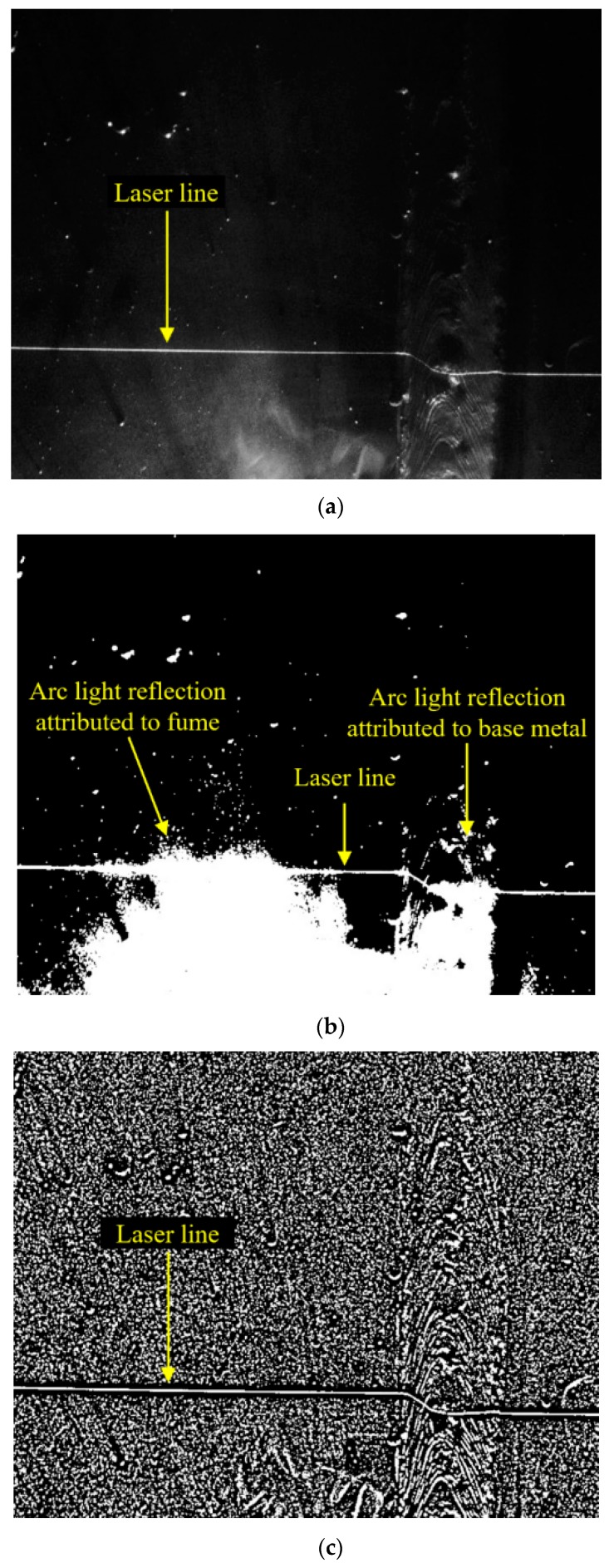
Thresholding operation used to identify the laser line. (**a**) Original image; (**b**) Image after global thresholding; (**c**) Image after adaptive thresholding.

**Figure 5:**
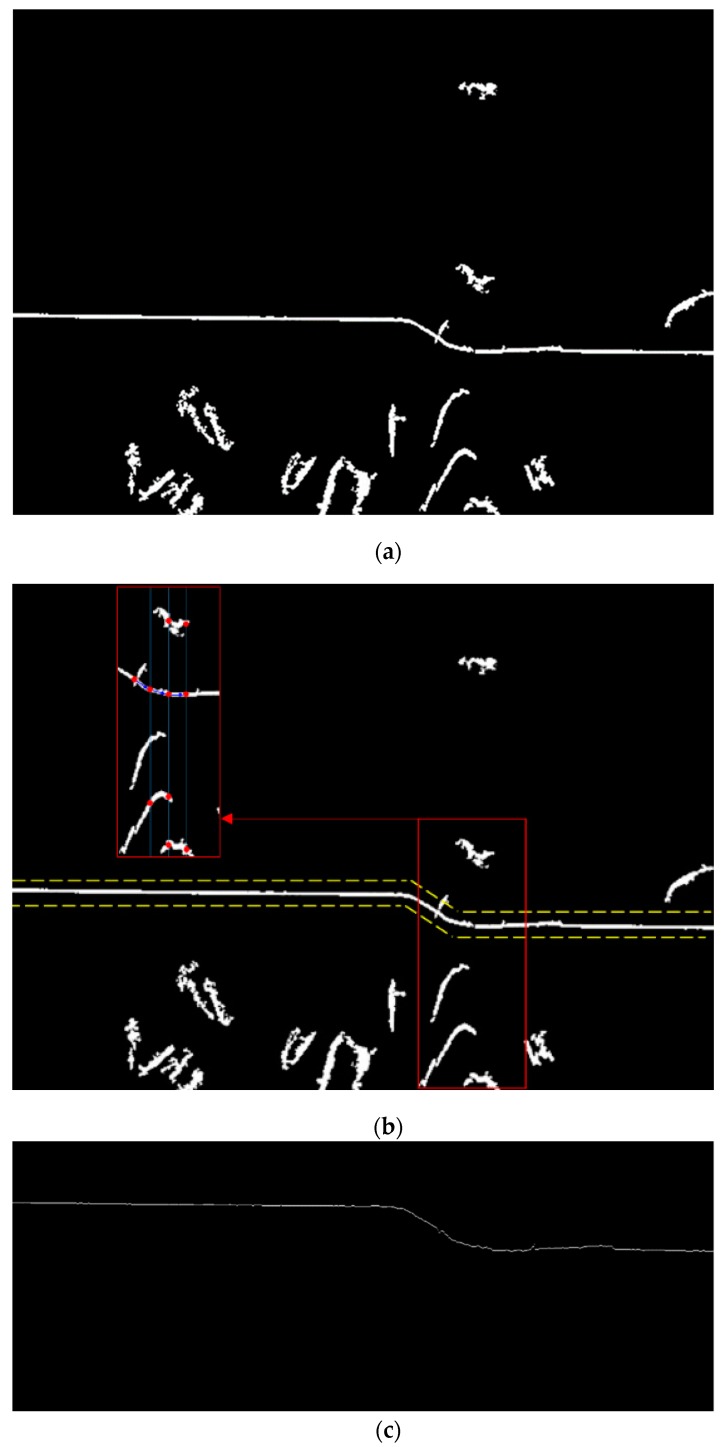
Contouring and thinning operations. (**a**) Contouring operation; (**b**) Thinning operation; (**c**) Resultant laser line after image processing.

To find the external bead shape, the original image (with a size of 2056 × 1524 pixels) acquired by the camera was sequentially processed in three steps: thresholding (binarization), contouring, and thinning. Given that global thresholding had trouble identifying the laser line due to the light reflection by welding fume and the base metal, adaptive thresholding [25] was used in this study (Figure 4). To set the adaptive threshold on a pixel-by-pixel basis, a weighted average of the 21 × 21 region was estimated around each pixel location, and a constant (C = 5) was subtracted. The average method was set to the Gaussian weighted average, and the parameters, including the block size and constant, were selected based on trial and error. Pixels larger than the threshold were set to 255, and the remaining pixels were set to zero. In the contouring step (white regions in Figure 4c, including the laser line) were separated based on the contouring operation, and a region with an area less than 1000 pixels^2^ was eliminated (Figure 5a). A thinning operation was used to remove the remaining noise pixels after the thresholding and contouring operations, and to extract the laser line. In the thinning step, all the column pixels were sequentially scanned on a pixel-by-pixel basis in the direction of the rows, and the continually connected white pixels, i.e., the white lines, were extracted in each column (Figure 5b). The center pixels of all the extracted white lines were then obtained. Subsequently, the extracted pixels on the laser line (obtained from a single line) and the remaining pixels that were not part of the laser line distributed randomly around the laser line. The laser line was acquired and was connected with the pixel of the column adjacent and closest to the current pixel (Figure 5c). After acquiring the laser line, we applied a subpixel detection method to improve the laser line detection accuracy and resolution in a direction perpendicular to the plane of the workpiece, as shown in Figure 3. As a result, a resolution of ~1.0 µm was achieved in a direction perpendicular to the plane of the workpiece. A resolution of ~20.0 µm was achieved in other directions on the plane of the workpiece, whereby the subpixel detection method was not used.

### 3.2. Camera Calibration

**Figure 6:**
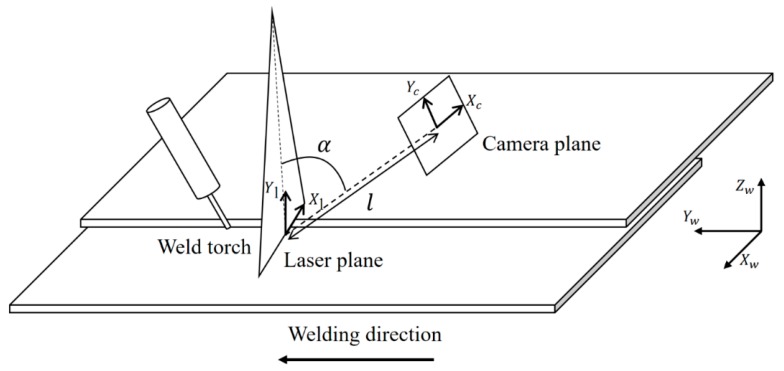
Coordinate systems of the laser vision system.

Figure 6 shows the coordinate systems associated with the LVS. The world coordinate system (weld coordinate system) is denoted as {*w*} with *X_w_*, *Y_w_*, and *Z_w_*, representing the three orthogonal directions, whereby *Y_w_* is the welding direction that originates at the starting point of the weld seam. The camera coordinate system is denoted as {*c*} with *X_c_*, *Y_c_*, and *Z_c_*, representing the three orthogonal directions, whereby *Z_c_* is assumed as the viewing direction of the camera along the optical axis. The laser coordinate plane is denoted as {*l*} with *X_l_* and *Y_l_* as the coordinates along with the two orthogonal, in-plane directions. A point on the laser line is denoted by (*u*, *v*) in the image plane.

To model the relationship between a point in the world coordinate and its projection point in the image plane, the camera was calibrated on the basis of the pinhole camera model [21], and the relationship is explained by Equations (1)–(5). The matrix **M** is the intrinsic parameter matrix of the camera and includes the magnification coefficients from the image plane to the image coordinates (*f_x_*, *f_y_*), the coordinates of the principal point of the camera (*c_x_*, *c_y_*), and the skew (*s* = 0 in this study). The joint rotation–translation matrix [**R**|**t**] is the extrinsic parameter matrix which defines the position of the camera center and the camera’s heading in the world coordinates, matrix **R** is a rotation matrix, and vector **t** is the origin of the world coordinate system in reference to the camera’s coordinate system. As shown in Figure 1, the relative position between the camera and the laser source is fixed in this study, and the rotation angle is also constant: $\theta_{x}=\alpha =45{}^{\circ}$, $\theta_{y}=\theta_{z}=0{}^{\circ}$. The matrix R|t was calculated based on a regression that was estimated with the use of a chessboard with a size of 3 × 3 mm.

(1) $$\left[\begin{array}{c} u\\ v\\ 1 \end{array}\right]=M[R|t]\left[\begin{array}{c} \begin{array}{c} X_{w}\\ Y_{w} \end{array}\\ Z_{w}\\ 1 \end{array}\right]=\left[\begin{array}{cc} \begin{array}{c} \begin{array}{cc} f_{x} & s\\ 0 & f_{y} \end{array}\\ \begin{array}{cc} 0 & 0 \end{array} \end{array} & \begin{array}{c} \begin{array}{c} c_{x}\\ c_{y} \end{array}\\ 1 \end{array} \end{array}\right]\left[\begin{array}{cc} \begin{array}{c} \begin{array}{cc} r_{11} & r_{12}\\ r_{21} & r_{22} \end{array}\\ \begin{array}{cc} r_{31} & r_{32} \end{array} \end{array} & \begin{array}{c} \begin{array}{cc} r_{13} & t_{1}\\ r_{23} & t_{2} \end{array}\\ \begin{array}{cc} r_{33} & t_{3} \end{array} \end{array} \end{array}\right]\left[\begin{array}{c} \begin{array}{c} X_{w}\\ Y_{w} \end{array}\\ Z_{w}\\ 1 \end{array}\right]$$

(2) $$R_{x}\left(\theta_{x}\right)=\left[\begin{array}{ccc} r_{11} & r_{12} & r_{13}\\ r_{21} & r_{22} & r_{23}\\ r_{31} & r_{32} & r_{33} \end{array}\right]=\left[\begin{array}{ccc} 1 & 0 & 0\\ 0 & \cos\theta_{x} & -\sin\theta_{x}\\ 0 & \sin\theta_{x} & \cos\theta_{x} \end{array}\right]$$

(3) $$R_{y}\left(\theta_{y}\right)=\left[\begin{array}{ccc} r_{11} & r_{12} & r_{13}\\ r_{21} & r_{22} & r_{23}\\ r_{31} & r_{32} & r_{33} \end{array}\right]=\left[\begin{array}{ccc} \cos\theta_{y} & 0 & \sin\theta_{y}\\ 0 & 1 & 0\\ -\sin\theta_{y} & 0 & \cos\theta_{y} \end{array}\right]$$

(4) $$R_{z}\left(\theta_{z}\right)=\left[\begin{array}{ccc} r_{11} & r_{12} & r_{13}\\ r_{21} & r_{22} & r_{23}\\ r_{31} & r_{32} & r_{33} \end{array}\right]=\left[\begin{array}{ccc} \cos\theta_{z} & -\sin\theta_{z} & 0\\ \sin\theta_{z} & \cos\theta_{z} & 0\\ 0 & 0 & 1 \end{array}\right]$$

(5) $$R=R_{x}\left(\theta_{x}\right)R_{y}\left(\theta_{y}\right)R_{z}\left(\theta_{z}\right)$$

The calibration based on the pinhole camera model was highly effective when the LVS was fixed (Figure 1). However, larger errors are generated when the LVS rotates, as indicated in Table 3. Moreover, the objective of this study was the development of the LVS that could respond to the welding robot movement and the measurement of the geometry of the weld bead in real-time. Therefore, the camera calibration used for the robot movement was added using calibration blocks in addition to the calibration based on the pinhole camera model.

Given that our LVS includes the 6-axis gyro sensor, the rotation angles, i.e., $\theta_{1},\;\;\theta_{2}$, and $\theta_{3}$, for the respective axes of *X_w_*, *Y_w_*, and *Z_w_*, of the LVS were measured in real-time. In the case where the *Y_w_* rotation errors existed (i.e., rotation errors around the welding direction), these errors were corrected based on the transformation of the rotation matrix (Equation (6)). Accordingly, the measured $\theta_{2}$ and Table 4 present the calibration results for the *Y_w_* rotation error.

(6) $$\left(\begin{array}{cc} cos\theta_{2} & -sin\theta_{2}\\ sin\theta_{2} & cos\theta_{2} \end{array}\right)\left(\begin{array}{c} x_{measrued}\\ y_{measured} \end{array}\right)=\left(\begin{array}{c} x_{corrected}\\ y_{corrected} \end{array}\right)$$

**Figure 7:**
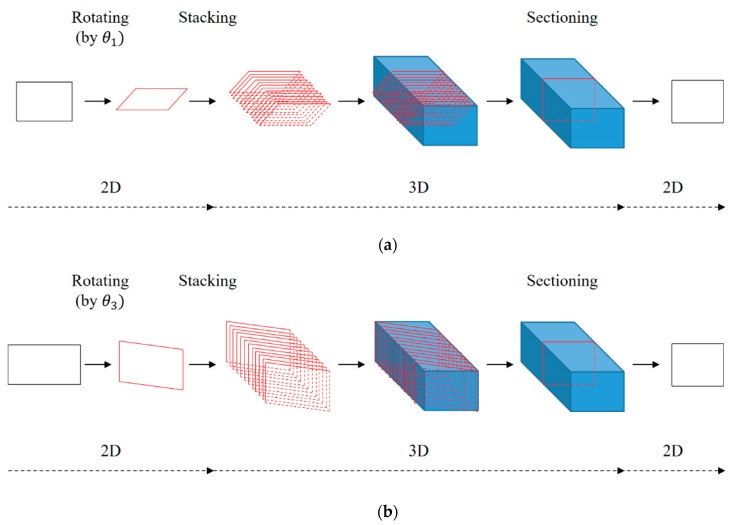
Calibration process for the (**a**) *X_w_* and (**b**) *Z_w_* rotation errors.

The *X_w_* and *Z_w_* rotation errors were corrected based on the same calibration process, which is depicted in Figure 7. Firstly, the two-dimensional (2D) geometry data that is measured from a laser stripe and calculated by Equations (1)–(5), and the rotation angles of the LVS from the six-axis gyro sensor are acquired at every sampling instance. Subsequently, this 2D plane is rotated by the acquired angles ($\theta_{1}$, $\theta_{3}$), and these rotated 2D planes, which are acquired sequentially every 0.01 ms, are stacked to restore the original 3D shape of the workpiece (a calibration block was used in this test). Lastly, the calibrated 2D plane is extracted every 0.1 ms by sectioning the 3D shape.

**Figure 8:**
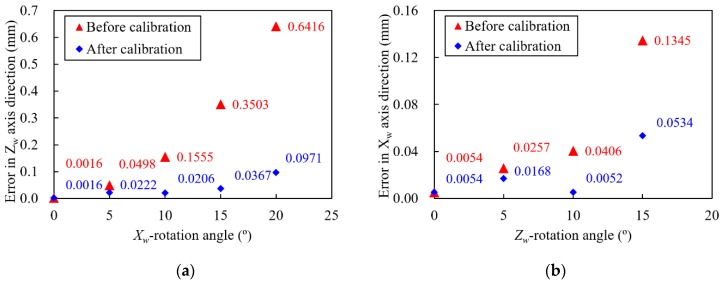
Calibration results of the (**a**) *X_w_* and (**b**) *Z_w_* rotation errors.

**Figure 9:**
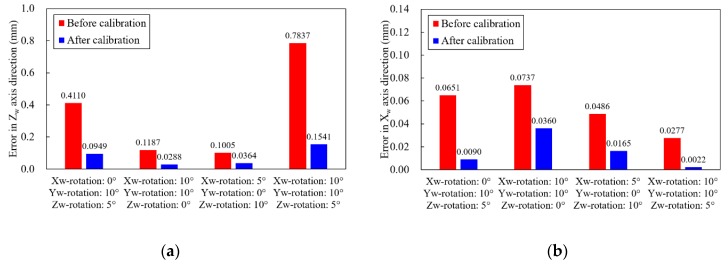
Measurement errors in the cases of complex rotation. (**a**) Errors in *Z_w_* axis direction; (**b**) Errors in *X_w_* axis direction.

This calibration method proposed in this study can reduce the errors generated by the arbitrary motions of the welding robot. Figure 8 shows the calibration results of the *X_w_* and the *Z_w_* rotation errors. By applying this calibration method, the average errors caused by the LVS rotation were reduced by 85.1% in the case of the *X_w_* rotation error, and by 60.8% in the case of the *Z_w_* rotation error. Complex rotation, which means simultaneous rotation with respect to the *X_w_*, *Y_w_*, and *Z_w_* axes, is the most common motions of the welding robots in assembly lines. Figure 9 shows the calibration results in the case of complex rotations. Each axis error was calibrated sequentially in this study in the order of *Y_w_*, *Z_w_*, and *X_w_*. The average errors caused by the LVS’s complex rotation were reduced by 77.8% for errors in the *Z_w_* axis direction, and by 70.4% for errors in the *X_w_* axis direction. It is evident that the calibration method proposed is highly effective in reducing the errors caused by the LVS’s complex motion.

### 3.3. Geometric Feature Extraction of Weld Joint

**Figure 10:**
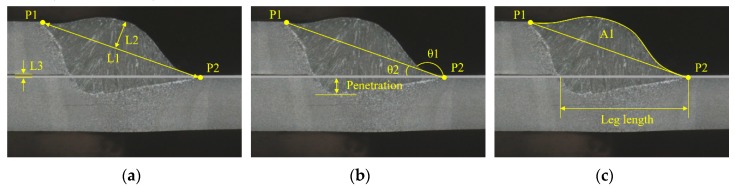
Weld bead parameter. (**a**) L1, L2, and L3; (**b**) θ1, θ2, and penetration; (**c**) A1 and leg length.

To acquire the geometric features of the weld joint, two feature points (P1 and P2) were extracted from the bead shape profile shown in Figure 10 from the image-processed laser line shown in Figure 5. Given that there is a significant geometric change in the boundary between the base metal and the weld, the two feature points on the boundary were determined based on the gradient of the laser line profile. The slope was calculated within intervals, which spanned 5, 10, 15, 20, and 25 pixels. Equation (7) was used for these calculations at every pixel of the laser line executed sequentially from one end of the laser line to the other end. If four of the five slopes at a certain pixel exceeded the threshold value of 0.15 mm, the pixel was determined as the feature point.

(7) $$Slope=\left|\frac{H(x+d)-H(x)}{L(x+d)-L(x)}\right|\;where\;d=5,\;10,\;15,\;20,\;25$$

where *d* is the pixel distance, and *L*(*x*) and *H*(*x*) are the horizontal and the vertical coordinates of the pixel, respectively. 

Figure 10 shows six geometric features of weld joints estimated by the two feature points. Accordingly, the following bead profiles are presented: the distance between the two points (L1), maximum distance between L1 and bead profile (L2), the gap between workpieces (L3), the tow angle (θ1), the angle between L1, and the upper line of the bottom plate (θ2), and the area between L1 and the external edge of the weld bead (A1). All geometric features except L3 are calculated by the two feature points. By contrast, the calculation of L3 needs the thickness of the upper plate, which is assumed to be known in this study. That is, L3 is calculated by subtracting the thickness of the upper plate from the difference between the two feature points in the perpendicular direction of the plates. Despite the fact that there are many specifications that define θ1, and despite the explanation of the conceptual method used to measure θ1, it is difficult to find specific guidelines on how to calculate θ1. This means that the measured value of θ1 can vary considerably depending on the measurement location and measurement method. Therefore, in this study, we applied linear regression using the coordinate values of the pixel near P2 to reduce the measurement error of θ1. Eleven pixels were selected along the external edge of the weld bead. These were 15 to 25 pixels apart from P2 in the horizontal direction from P2 to P1. A linear regression equation was estimated using the coordinates of the 11 pixels on the external edge. Lastly, θ1 was calculated based on the inverse sine of the slope of the linear equation. Figure 10 also depicts the parameters of the internal bead shape, penetration, and leg length. The six geometric features are calculated in real-time during the welding process and are used for estimating the penetration, leg length, and tensile strength. The developed LVS system measured the external bead shape 100 times per second. With the exception of the outliers, the acquired data were averaged at five intervals, and the averaged data were displayed, stored in memory, and used by the DNN models.

### 3.4. Real-Time Implementation and Verification of the LVS

**Figure 11:**
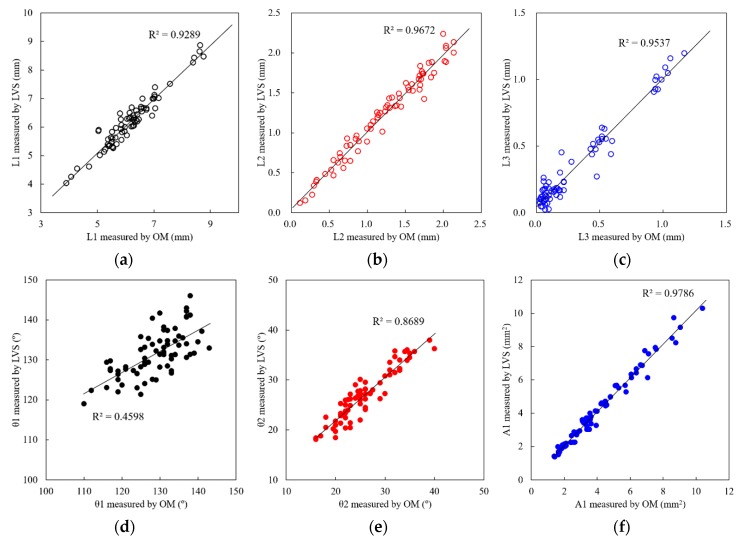
Comparison between LVS and optical microscopic (OM) measurements: (**a**) L1, (**b**) L2, (**c**) L3, (**d**) θ1, (**e**) θ2, and (**f**) A1.

The developed LVS was implemented on a GMAW torch, and the sensor’s real-time measurement performance was tested during the GMAW process. The measurement accuracy of the LVS was evaluated by comparing the six geometric features acquired from the LVS with those from weld cross-section examinations. Figure 11 compares the results measured by the LVS and an optical microscope (OM), and Table 5 presents the average errors of all the geometric features. All geometric features except θ1 have R^2^ values greater than 0.86, and the R^2^ of θ1 was approximately 0.46. This value is particularly low, and the linear relation is not proper for θ1. This result is thought to be caused by the measurement method that is not exactly specific to θ1, as described in Section 3.3. That is, the difference between the measured OM and LVS values is quite large compared with the other geometric features. Conversely, the average error for θ1 is quite small because the differences between the OM and LVS measurements in Figure 11d are quite small compared with the value of θ1. The average errors of all the geometric features were less than 8.0%. It is considered that this result is equivalent to or better than the results of previous research studies [21], despite the fact that the joint shapes tested by the LVS are different with respect to each other.

## 4. Prediction Models for the Estimation of Penetration, Leg Length, and Tensile Strength

### 4.1. DNN Models

**Figure 12:**
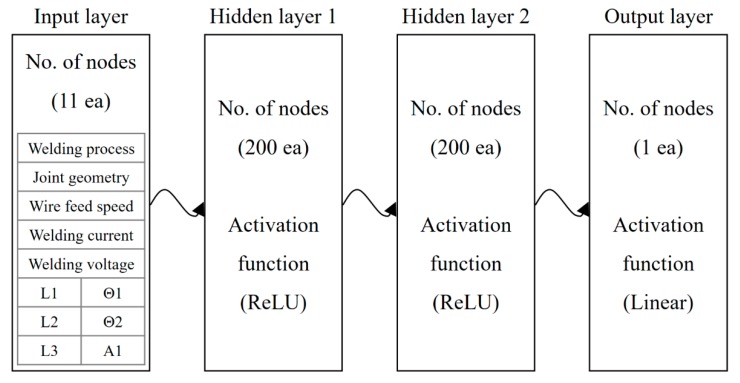
Structure of the DNN models for penetration, leg length, and tensile strength.

To predict tensile strength and the internal bead shape, i.e., penetration and leg length that considerably affect weld quality, a DNN model was applied as the prediction model. As shown in Figure 12, we modeled our neural architecture as a two-hidden-layer DNN and assigned 200 neurons to each of these hidden layers. The neurons of every preceding layer were fully connected to those of the succeeding layer. The inputs to this neural network are the five welding process parameters and the six geometric features of the weld joint described in Section 3.3. These welding process parameters corresponded to different (a) types of welding processes (CMT, DC standard), (b) welding positions (PA, PC), (c) wire feeding speeds, (d) currents, and (e) voltages. All the hidden layers of this architecture utilized rectified linear unit (ReLU) functions to calculate their respective intermediate outputs. The output layer had only one node and utilized a linear function as the activation function. Therefore, our models produced real-valued numbers to estimate the outputs. Given that the outputs of our DNN model included the penetration, leg length, and tensile strength of the weld joint, three separate DNN models with the same architecture were estimated in this study. To generate the DNN model, 189 training sets, 28 validation sets, and 29 test sets were used for each model. Each dataset contained 11 input variables (Figure 12) and an output variable that corresponded to one of the three DNN models. The backpropagation algorithms were carried out with batch sizes with 10–500 epochs. Table 6 presents the training results of the three DNN models. For the leg length and tensile strength, all the errors of the models—including the training, validation, test, and total errors—were less than 6.29 %. The reason for which the training errors of the penetration model were relatively large is attributed to the increased deviation of the penetration measurement.

### 4.2. Verification of Prediction Models

**Figure 13:**
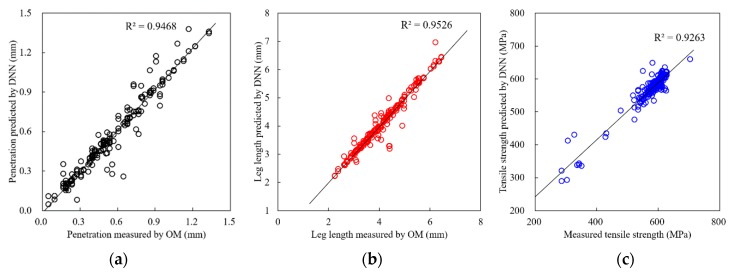
Verification of DNN prediction models for (**a**) penetration, (**b**) leg length, and (**c**) tensile strength.

Figure 13 shows the verification results of the prediction models for penetration, leg length, and tensile strength. For all models, the relationship between the predicted value by the DNN model and the measured value is almost linear. The coefficient of determinations (*R*^2^) of all model were larger than 0.92. This means that each prediction model can make predictions for more than 92% of the experimental data, and cannot explain the total variations for less than 8% of the remaining data. It is thought that the models yielded equivalent or improved prediction performances compared with previous studies [10,26], despite the fact that the experimental setup—including materials, welding condition, and others—were different. Therefore, it is considered that the developed models have increased predictability for their output parameters. For tensile strength, data can be divided in two regions according to the fracture mode in tensile testing. The group associated with tensile strength data in the range of 550 to 650 MPa was obtained when fractures occurred at the base metal during tensile testing, including the heat-affected zone. This group was classified to have an acceptable weld quality, and most of the data points were concentrated in this group. The other group was associated with tensile strength data smaller than approximately 550 MPa when the fracture during tensile testing occurred at the weld metal. This fracture mode was generally classified as an unacceptable weld quality outcome. Differences in the fracture mode were mainly caused by the weld size measured by the leg length and penetration. That is, when the weld size was larger than a specific value, the fracture region developed during the tensile testing abruptly changed from the welded region to the base metal region (SPFH590 in the study). This phenomenon caused a sudden change in tensile strength with changes in the fracture mode. Owing to the experimental setup, including the workpiece (SPFH with a thickness of 2.3 mm) and welding wire (ER70S), most of the data were concentrated in the former group at the welding condition used in this study (Table 2). Considering the penetration and leg length, most of the fractures occurred in the base metals when the penetration and leg length were less than approximately 0.3 mm and 3.0 mm, respectively. This may be responsible for the minor decrease in the *R*^2^ values of the tensile strength model. 

## 5. Conclusions

In this study, we developed an LVS that can measure the external bead shape and can significantly reduce the measurement error caused by the complex movement of the welding robot. Prediction models for the leg length and the penetration of welds were also estimated using DNN. By integrating the LVS and the prediction models with operating software, a graphical-user-interface-based laser vision system was developed for real-time applications. Notable developments and outcomes from this study follow.

- First, we proposed a three-step image processing algorithm which consisted of thresholding, contouring, and thinning to identify the weld bead profile
- Second, we proposed a camera calibration method that could considerably reduce measurement errors generated by the arbitrary 3D rotations of the LVS, which was installed at the welding robot. This method extracted more accurate weld bead profiles based on the rotations of the measured image data or the rotations and sectioned image data with the use of the rotation angle of the LVS measured by a six-axis gyro sensor
- We also developed DNN models that could predict penetration, leg length, and tensile strength, at different welding process parameters. Accordingly, the geometrical features were measured by the LVS. The *R*^2^ values of all the prediction models were > 0.92

The results of this study substantially contribute to the state of knowledge regarding the automation of the welding process and NDT technology for arc welding processes. Our research is of particular interest and use to the automotive industry because of (a) the enhanced understanding of the laser vision system used for the inspection of the weld quality, and (b) the potential for direct improvement to real-time NDT technology. Despite our study’s contributions, some limitations are worth noting. Although the prediction models of our laser vision system effectively estimate internal weld bead parameters, the application of the models is limited to the scope of this study. Future work will focus on the collection of more database entries pertaining to the various materials and welding conditions and on the expansion of the prediction models. 

## Figures and Tables

**Table 1:** Specification of the test material.

Material	Thickness (mm)	Tensile Strength (MPa)	Chemical Composition (wt%)					
			C	Si	Mn	P	S	Fe
SPFH590	2.3	590	0.07	0.14	1.44	0.013	0.002	Bal.

**Table 2:** Welding conditions.

Welding Process	CMT	DC Standard
Wire feeding speed [current/voltage] (m/min, A, V)	3.0 [115 A/13.3 V]	3.0 [132 A/16.6 V]
	4.0 [139 A/14.3 V]	4.0 [162 A/17.7 V]
	5.0 [165 A/15.2 V]	5.0 [186 A/19.1 V]
	6.0 [195 A/15.5 V]	6.0 [217 A/21.0 V]
	7.0 [214 A/16.2 V]	7.0 [238 A/23.2 V]
Welding position	Flat position (PA), horizontal position (PC)	
Gap (mm)	0, 0.1, 0.2, 0.5, 1.0	
Welding speed (cm/min)	100	
CTWD (mm)	15	
Work angle (°)	45	
Travel angle (°)	0	

**Table 3:** Errors caused by LVS rotations.

Item	*Y_w_* Rotation Error (*Y_w_*: Welding Direction)	*X_w_* Rotation Error	*Z_w_* Rotation Error
LVSmovement	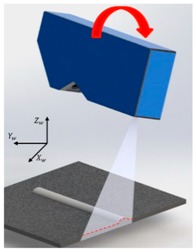	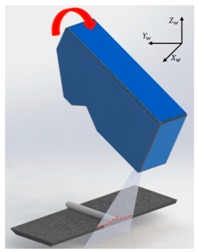	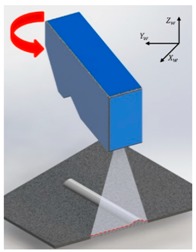
Error in a bead shape	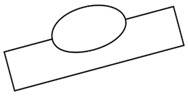	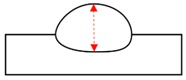	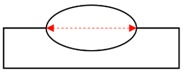
Description	Distortion in weld position	Distortion in bead height	Distortion in bead width

**Table 4:** Calibration results for the *Y_w_* rotation error.

$Y_{w}\;\mathrm{Rotation}\;\mathrm{Angle}\;(\theta_{2})$	Measured Data	Calibrated Data
2.5°	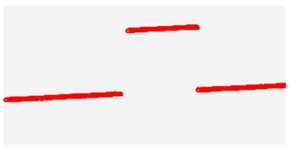	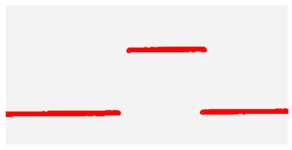
5.0°	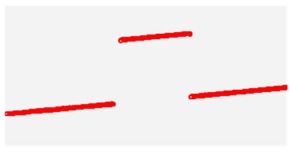	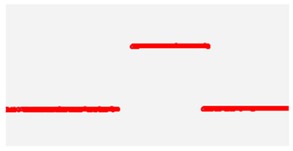
10.0°	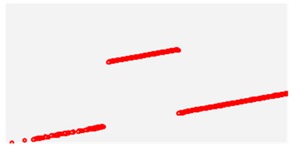	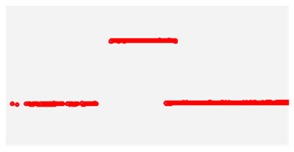

**Table 5:** Average errors of the various geometric features.

Feature	Average Error (%)
L1	3.2
L2	7.5
L3	2.2
θ1	4.0
θ2	8.0
A1	6.2

**Table 6:** Training results of the DNN models.

Item	Penetration Model	Leg Length Model	Tensile Strength Model
Training error (%)	5.55	1.05	2.64
Validation error (%)	14.98	6.29	3.95
Test error (%)	12.04	6.03	4.42
Total error (%)	7.89	2.59	3.11
